# Evaluation of the awareness of novel advanced therapies among family medicine residents in Spain

**DOI:** 10.1371/journal.pone.0214950

**Published:** 2019-04-03

**Authors:** Miguel Sola, Carmen Sanchez-Quevedo, Miguel A. Martin-Piedra, Victor Carriel, Ingrid Garzon, Jesus Chato-Astrain, Oscar-Dario Garcia-Garcia, Miguel Alaminos, Fernando Campos

**Affiliations:** 1 Family Medicine Unit, School of Medicine, University of Granada, Granada, Spain; 2 Instituto de Investigación Biosanitaria ibs.GRANADA, Granada, Spain; 3 Department of Histology (Tissue Engineering Group), School of Medicine, University of Granada, Granada, Spain; Stellenbosch University Faculty of Medicine and Health Sciences, SOUTH AFRICA

## Abstract

**Background:**

Advanced therapies are increasingly demanded by patients with the intent of treating some incurable conditions. Because family medicine professionals play an important role as health educators, their residency programs should incorporate new knowledge related to advanced therapies. To successfully implement these programs, how family medicine residents perceive these therapies should be investigated. The main components of perception, i.e. conceptual, procedural and attitudinal, refer to knowledge, skills and feelings, respectively.

**Methods and findings:**

We designed a specific questionnaire to assess the components of perceptions of advanced therapies in 300 medical residents enrolled in the Spanish National Family Medicine Residency Program. Each component consisted of 4 or 5 topics and each topic contained 6 items. Respondents scored highest in the procedural component (average 4.12±1.00), followed by the attitudinal (3.94±1.07) and conceptual component (3.04±1.43). Differences among the three components were statistically significant (p<0.00017). Family medicine residents perceived that procedures to implement advanced therapies are well established, especially their application. However, they felt their cognitive background was insufficient to respond efficiently to the expectations generated by these new therapeutic tools, especially in the regulatory framework. High awareness of the risks and limitations of these treatments was reflected by residents’ preference for clinically tested therapies. Although they appropriately situated treatment with these therapies within hospital care, they associated the biofabrication of novel products with research centers, although these therapeutic tools can be produced in different facilities.

**Conclusions:**

These results are potentially useful for designing future training programs and health policies for family medicine residents, and suggest the need to implement specific training programs in advanced therapies at the conceptual, procedural and attitudinal level.

## Introduction

In recent decades, genes, cells and tissues have been adapted as new therapeutic tools in medicine. In this new approach, known as advanced therapies, each of these therapeutic agents is termed an advanced therapy medicinal product (ATMP) [1]. As in other medicinal products such as drugs, devices and biological agents, products based on genes, cells and tissues are subject to regulatory requirements that vary widely among countries and product types [2]. The regulatory requirements for ATMPs were established in the European Union by two European Directives (2003/63/EC and 2009/120/EC) and by EC Regulation No. 1394/2007 of the European Parliament and Council. Specific regulations have also been established in different countries, e.g. FDA regulation in the USA [3]. In European countries, marketing authorization must follow a centralized procedure at the European Medicines Agency (EMA), and very precise guidelines must be met for product safety, control of the manufacturing process, and clinical trials [1].

Although these therapies may have great potential, their results thus far have not advanced as much as initially foreseen [4]. Although some scientifically- and clinically-proven gene, stem-cell and artificial tissue-based interventions are being successfully applied, unproven interventions with these therapies are also being sought and used by patients with the intent of treating some degenerative or incurable conditions [5, 6].The consequence is a growing international market in this field, with clinics around the world offering unproven and unapproved cell and tissue therapies for a vast array of conditions without evidence of safety or efficacy for these products. This phenomenon, generally known as ‘‘stem cell tourism”, is a subject of great debate and concern [7–10].

Among the concerns associated with the stem cell tourism market are physical and financial risks for patient and reputational risks for legitimate research on and clinical applications of advanced therapies [5, 11]. A further financial aspect with future implications is when patients return from receiving unproven and unapproved treatments in the private market and then demand follow-up care in publicly funded medical systems [5, 12]. As pointed out by Gunter and colleagues [9], patients need to be equipped to understand the difference between (a) formal clinical trials and the innovative practice of medicine (where their rights are protected and risks are controlled and communicated) and (b) fraudulent cell and tissue therapy practices (where there is no protection or demonstration of competency, and misinformation is the rule).

In this context, health education plays a fundamental role not only as an instrument for information and health promotion, but also as a necessary mechanism to guide patients towards the best choice, and thus to contribute to the sustainability of the health system. This is important because of the high costs involved in the implementation of advanced therapy programs in health systems [13]. The role of family medicine physicians in this educational process is highly relevant because they are the basic agents in the interrelation between patients and the health systems in all aspects related to scientific knowledge and their clinical condition. As has been clearly established, family medicine physicians must use data to monitor and manage their patient population, and use well-established science and knowledge to prioritize the clinical services that are most likely to benefit patient health. In addition, patients also expect their family medicine physician to help them prevent, understand and manage their diseases [14].

To equip family medicine physicians to carry out these activities, and therefore to prevent the potential risks of stem cell tourism, residency training programs should incorporate new knowledge and evidence related to advanced therapies, not only regarding the conceptual and procedural components of ATMPs, but also regarding physicians’ attitudinal approach.

In the present study we investigate different components of perception (e.g. conceptual, procedural and attitudinal) in family medicine residents in Spain in order to determine the variables or constructs which could serve as a foundation for their future learning processes in advanced therapies [15, 16]. These variables or constructs can be defined as the way in which students conceptualize and relate to the learning process, which is assumed to affect their learning and achievements [17]. Insights into residents’ perceptions will help us to understand their constructs, especially concerning their expectations with regard to the tasks they should learn about and become skilled in. The research reported here was designed to answer to the following question: what are the conceptual, procedural and attitudinal components perceived by family medicine residents regarding treatment with advanced therapies? The results are potentially useful for designing future training programs and health policies in family medicine.

## Materials and methods

### Sample

The present study was carried out at 20 hospitals and health centers operated by the Public Andalusian Health System (Servicio Andaluz de Salud) and accredited as training centers for family medicine residents. A total of 323 medical residents enrolled in the National Family Medicine Residency Program were invited to participate in the study, whose participation was voluntary. Altogether, 300 (92.9%) of the residents contacted agreed to be included in the study. Average age of the participants was 28.16±4.94 years. Slightly more than one fourth of the participants (84, 28%) were men (average age 28.81±6.11 years) and 216 (72%) were women (average age 27.91±4.38); these numbers are representative of the whole population of family medicine residents in Spain.

All participants signed an informed consent form, and all results were analyzed anonymously. The study and the protocol were approved by the Ethics and Research Committee of the University of Granada (ref. 62/CEIH/2016).

### Instrument

To evaluate family medicine residents’ perceptions regarding advanced therapies, we designed a specific questionnaire to solicit information on conceptual, procedural and attitudinal components. The conceptual component refers to knowledge (as information recall and remembering) of classifications and categories, principles and generalizations, theories, models and structures [18]. The procedural component reflects knowledge of subject-specific skills and algorithms, subject-specific techniques and methods, and criteria for determining when to use appropriate procedures [18]. The attitudinal component refers to a set of emotions and feelings experienced over time on a specific issue [19]. Each component consisted of 4 or 5 topics and each topic contained 6 items (Table 1). Residents rated each item on a five-point Likert-like scale: 1 = strongly disagree, 2 = disagree, 3 = neither agree nor disagree, 4 = agree and 5 = strongly agree (S1 Table). The questionnaire was given to the residents along with a brief explanation on the purpose of the instrument and instructions about how to complete the questionnaire.

**Table 1:** Items in the questionnaire corresponding to different components and topics. For each item, both the full question and its abbreviated item name are shown.

COMPONENT	TOPIC	ITEM	ITEM
**CONCEPTUAL**	**Advanced therapies**	**AdT1**	**Do you know what advanced therapies are?**
		**AdT2**	**Are you familiar with the concept of gene therapy?**
		**AdT3**	**Are you familiar with the concept of somatic cell therapy?**
		**AdT4**	**Are you familiar with the concept of combined advanced therapy?**
		**AdT5**	**Do you know if gene therapy is an advanced therapy?**
		**AdT6**	**Do you know if tissue engineering is an advanced therapy?**
	**Artificial tissues**	**ArT1**	**Are you familiar with the concept of artificial tissue?**
		**ArT2**	**Are you familiar with the concept of biomaterial?**
		**ArT3**	**Are you familiar with the concept of growth factors?**
		**ArT4**	**Do you distinguish conceptually between a natural tissue and an artificial one?**
		**ArT5**	**Are you familiar with the concept of tissue engineering?**
		**ArT6**	**Are you familiar with the concept of regenerative medicine?**
	**Cell and tissue basis of the human body**	**CTB1**	**Are you familiar with the concept of cell?**
		**CTB2**	**Are you familiar with the concept of tissue?**
		**CTB3**	**Are you familiar with the concept of stem cell?**
		**CTB4**	**Are you familiar with the concept of embryonic stem cell?**
		**CTB5**	**Are you familiar with the concept of adult stem cell?**
		**CTB6**	**Are you familiar with the concept of IPS cell?**
	**Novel medical products**	**NMP1**	**Do you know if a cell can be considered a medicine?**
		**NMP2**	**Do you know if a tissue can be considered a medicine?**
		**NMP3**	**Do you know if transplanted organs are medicines?**
		**NMP4**	**Do you know if biomaterials are used to treat diseases?**
		**NMP5**	**Do you know if growth factors are used to treat diseases?**
		**NMP6**	**Do you know if there are benefits of these therapies with respect to current treatment techniques?**
	**Regulatory framework**	**RF1**	**Do you know if there is specific EU legislation for advanced therapies?**
		**RF2**	**Do you know what GMP rooms are?**
		**RF3**	**Do you know if it is mandatory to manufacture advanced therapy products considered medicines in GMP rooms?**
		**RF4**	**Do you know if it is mandatory to perform a clinical trial before using advanced therapy products?**
		**RF5**	**Do you know if all advanced therapies require authorization from the Spanish and European agencies for their implementation?**
		**RF6**	**Do you know if advanced therapies are in the service portfolio of the National Health System?**
**PROCEDURAL**	**Application and use of advanced therapies**	**AUAT1**	**Would you use the patient's own cells for treatment with cell therapy?**
		**AUAT2**	**Would you use cells from donors to treat a patient with cell therapy?**
		**AUAT3**	**Would you apply cell therapy to treat a disease?**
		**AUAT4**	**Would you apply gene therapy to treat a disease?**
		**AUAT5**	**Would you apply tissue-engineered tissues to treat a disease?**
		**AUAT6**	**Would you apply artificial tissues built with cells, biomaterials and growth factors together to treat a disease?**
	**Application center for advanced therapies**	**ACAT1**	**Would you use hospitals for a cell therapy treatment?**
		**ACAT2**	**Would you use primary care health centers to monitor patients treated with cell therapy?**
		**ACAT3**	**Would you use hospitals for a gene therapy treatment?**
		**ACAT4**	**Would you use primary care health centers to follow up patients treated with gene therapy?**
		**ACAT5**	**Would you use hospitals to treat a patient with artificial tissues generated by tissue engineering?**
		**ACAT6**	**Would you use primary care health centers to monitor patients treated with artificial tissues generated by tissue engineering?**
	**Biofabrication components for advanced therapies**	**BCAT1**	**Would you use umbilical cord stem cells to build artificial tissues?**
		**BCAT2**	**Would you use bone marrow stem cells to build artificial tissues?**
		**BCAT3**	**Would you use adipose tissue stem cells to build artificial tissues?**
		**BCAT4**	**Would you use dental pulp stem cells to build artificial tissues?**
		**BCAT5**	**Would you build artificial tissues with biomaterials?**
		**BCAT6**	**Would you build artificial tissues with growth factors?**
	**Centers for biofabrication and storage of advanced therapies**	**CBSAT1**	**Would you store artificial tissues in tissue banks for deferred use?**
		**CBSAT2**	**Would you store cells in tissue banks for deferred use?**
		**CBSAT3**	**Would you store genes in tissue banks for deferred use?**
		**CBSAT4**	**Would you use a primary care health center to build artificial tissues?**
		**CBSAT5**	**Would you use a pharmaceutical company to build artificial tissues?**
		**CBSAT6**	**Would you use a research center to build artificial tissues?**
**ATTITUDINAL**	**Research interest in advanced therapies**	**RIAT1**	**Are you interested in cell therapy research?**
		**RIAT2**	**Are you interested in gene therapy research?**
		**RIAT3**	**Are you interested in artificial tissue therapy research?**
		**RIAT4**	**Do you think clinical trials in cell therapy are a good idea?**
		**RIAT5**	**Do you think clinical trials with artificial tissues are a good idea?**
		**RIAT6**	**Do you think clinical trials in gene therapy are a good idea?**
	**Research interest in classical therapies**	**RICT1**	**Are you interested in research in surgery?**
		**RICT2**	**Are you interested in pharmacotherapy research?**
		**RICT3**	**Are you interested in research in physical medicine and physiotherapy?**
		**RICT4**	**Are you interested in psychotherapy research?**
		**RICT5**	**Do you think clinical trials to test pharmaceutical drugs are a good idea?**
		**RICT6**	**Do you think clinical trials in physical therapy are a good idea?**
	**Valuation of centers for advanced therapies**	**VCAT1**	**Do you prefer hospitals for the application of cell therapy?**
		**VCAT2**	**Do you prefer hospitals for the application of gene therapy?**
		**VCAT3**	**Do you prefer hospitals for the application of artificial tissue therapy?**
		**VCAT4**	**Do you prefer hospitals for the application of combined advanced therapies?**
		**VCAT5**	**Do you prefer artificial tissues to be manufactured in hospitals?**
		**VCAT6**	**Do you prefer artificial tissues to be manufactured by the pharmaceutical industry?**
	**Valuation of treatment with advanced therapies**	**VTAT1**	**Do you think cell therapy is a good idea?**
		**VTAT2**	**Do you think gene therapy is a good idea?**
		**VTAT3**	**Do you think therapy with artificial tissues is a good idea?**
		**VTAT4**	**Do you think so-called advanced therapies is a good idea?**
		**VTAT5**	**Do you think therapy with physical medicine is a good idea?**
		**VTAT6**	**Do you think pharmaceutical drug therapy is a good idea?**

The set of topics and items included in the questionnaire was initially selected by the authors of the present work, who have a background in advanced therapies and family medicine. To validate the questionnaire prior to its use, the instrument was critically analyzed by a panel of national and international experts in the field. After that, a pilot study was carried out in a group of 30 residents who volunteered to complete the questionnaire, and its internal consistency and reliability were found to be very good (Cronbach’s alpha index of 0.9571). After this preliminary process, the questionnaire was used for the whole study sample. Analysis of the results obtained from 300 residents confirmed the validity of the preliminary results, as determined by a Kaiser-Meyer-Olkin measure of sampling adequacy (KMO) value of 0.8939, a Bartlett's test of sphericity value of 0.0000, and a Cronbach’s alpha index of 0.9561.

### Statistical analysis

Average values and standard deviations were calculated for each item, for each topic and for each component for male and female residents separately and for the entire sample together. To identify statistically significant differences between groups, we used ANOVA. This analysis was used to carry out pairwise comparisons of the following groups: 1) components of perception, 2) topics within the same component, 3) items within the same topic, 4) male and female residents. All statistical analyses were two-tailed. To correct for multiple testing, a Bonferroni-adjusted p value below 0.00017 was considered statistically significant.

## Results

The results for each component, topic and item are summarized in Tables 2, 3 and 4 and raw data are available in the S1 File.

**Table 2:** Mean values and standard deviations (SD) for the conceptual component and for each topic and each item included in this component. In each case, results are shown for male and female residents separately and for all residents together. The last column shows the statistical p value for ANOVA comparisons between genders.

COMPONENT	TOPIC	ITEM	MEAN MALES	SD MALES	MEAN FEMALES	SD FEMALES	MEAL ALL RESIDENTS	SD ALL RESIDENTS	MALES VS. FEMALES ANOVA p VALUE
**CONCEPTUAL**	**Advanced therapies**	**AdT1**	**2.86**	**1.31**	**2.68**	**1.20**	**2.73**	**1.23**	**0.252**
		**AdT2**	**3.71**	**1.06**	**3.39**	**1.18**	**3.48**	**1.16**	**0.031**
		**AdT3**	**2.54**	**1.23**	**2.16**	**1.14**	**2.26**	**1.18**	**0.012**
		**AdT4**	**2.27**	**1.20**	**2.15**	**1.09**	**2.18**	**1.12**	**0.424**
		**AdT5**	**2.77**	**1.33**	**2.72**	**1.34**	**2.74**	**1.33**	**0.764**
		**AdT6**	**2.68**	**1.27**	**2.77**	**1.33**	**2.74**	**1.32**	**0.596**
		**ALL**	**2.81**	**1.31**	**2.64**	**1.29**	**2.69**	**1.29**	**0.019**
	**Artificial tissues**	**ArT1**	**3.46**	**1.22**	**3.56**	**1.20**	**3.53**	**1.20**	**0.556**
		**ArT2**	**3.23**	**1.26**	**3.13**	**1.23**	**3.15**	**1.24**	**0.526**
		**ArT3**	**4.07**	**0.90**	**3.88**	**0.98**	**3.93**	**0.96**	**0.121**
		**ArT4**	**3.58**	**1.08**	**3.51**	**1.16**	**3.53**	**1.14**	**0.636**
		**ArT5**	**3.07**	**1.26**	**3.10**	**1.22**	**3.09**	**1.23**	**0.871**
		**ArT6**	**3.36**	**1.24**	**3.09**	**1.23**	**3.16**	**1.24**	**0.090**
		**ALL**	**3.46**	**1.20**	**3.38**	**1.21**	**3.40**	**1.21**	**0.176**
	**Cell and tissue basis of the human body**	**CTB1**	**4.55**	**0.72**	**4.65**	**0.68**	**4.62**	**0.69**	**0.258**
		**CTB2**	**4.54**	**0.68**	**4.64**	**0.65**	**4.61**	**0.66**	**0.223**
		**CTB3**	**4.44**	**0.83**	**4.52**	**0.74**	**4.50**	**0.77**	**0.429**
		**CTB4**	**4.04**	**1.05**	**3.97**	**1.05**	**3.99**	**1.05**	**0.638**
		**CTB5**	**3.68**	**1.26**	**3.58**	**1.14**	**3.61**	**1.17**	**0.529**
		**CTB6**	**2.00**	**1.18**	**1.79**	**1.07**	**1.85**	**1.10**	**0.134**
		**ALL**	**3.87**	**1.32**	**3.86**	**1.35**	**3.86**	**1.34**	**0.830**
	**Novel medical products**	**NMP1**	**2.95**	**1.21**	**3.09**	**1.25**	**3.05**	**1.24**	**0.379**
		**NMP2**	**3.02**	**1.23**	**3.06**	**1.25**	**3.05**	**1.24**	**0.843**
		**NMP3**	**2.87**	**1.27**	**2.76**	**1.24**	**2.79**	**1.24**	**0.512**
		**NMP4**	**3.63**	**1.20**	**3.37**	**1.29**	**3.44**	**1.27**	**0.110**
		**NMP5**	**4.11**	**0.96**	**4.06**	**1.09**	**4.08**	**1.06**	**0.756**
		**NMP6**	**2.60**	**1.35**	**2.67**	**1.26**	**2.65**	**1.28**	**0.666**
		**ALL**	**3.20**	**1.31**	**3.17**	**1.31**	**3.18**	**1.31**	**0.690**
	**Regulatory framework**	**RF1**	**1.98**	**1.20**	**1.99**	**1.17**	**1.99**	**1.18**	**0.924**
		**RF2**	**1.62**	**1.00**	**1.34**	**0.77**	**1.42**	**0.85**	**0.010**
		**RF3**	**1.61**	**0.98**	**1.31**	**0.72**	**1.40**	**0.81**	**0.005**
		**RF4**	**2.82**	**1.41**	**2.77**	**1.41**	**2.78**	**1.41**	**0.771**
		**RF5**	**2.65**	**1.41**	**2.57**	**1.38**	**2.60**	**1.39**	**0.652**
		**RF6**	**2.24**	**1.26**	**2.06**	**1.18**	**2.11**	**1.20**	**0.263**
		**ALL**	**2.15**	**1.30**	**2.01**	**1.26**	**2.05**	**1.28**	**0.034**
	**CONCEPTUAL COMPONENT**	** **	**3.10**	**1.42**	**3.01**	**1.43**	**3.04**	**1.43**	**0.009**

**Table 3:** Mean values and standard deviations (SD) for the procedural component and for each topic and each item included in this component. In each case, results are shown for male and female residents separately and for all residents together. The last column shows the statistical p value for ANOVA comparisons between genders.

COMPONENT	TOPIC	ITEM	MEAN MALES	SD MALES	MEAN FEMALES	SD FEMALES	MEAL ALL RESIDENTS	SD ALL RESIDENTS	MALES VS. FEMALES ANOVA p VALUE
**PROCEDURAL**	**Application and use of advanced therapies**	**AUAT1**	**4.42**	**0.98**	**4.38**	**0.77**	**4.39**	**0.84**	**0.699**
		**AUAT2**	**3.95**	**1.21**	**3.93**	**0.96**	**3.93**	**1.03**	**0.842**
		**AUAT3**	**4.32**	**0.82**	**4.27**	**0.80**	**4.28**	**0.80**	**0.609**
		**AUAT4**	**4.38**	**0.85**	**4.24**	**0.82**	**4.28**	**0.83**	**0.173**
		**AUAT5**	**4.43**	**0.80**	**4.28**	**0.75**	**4.32**	**0.77**	**0.126**
		**AUAT6**	**4.17**	**0.94**	**4.10**	**0.94**	**4.12**	**0.94**	**0.592**
		**ALL**	**4.28**	**0.96**	**4.20**	**0.85**	**4.22**	**0.88**	**0.100**
	**Application center for advanced therapies**	**ACAT1**	**4.33**	**0.83**	**4.26**	**0.75**	**4.28**	**0.77**	**0.486**
		**ACAT2**	**4.04**	**1.21**	**3.93**	**1.07**	**3.96**	**1.11**	**0.462**
		**ACAT3**	**4.33**	**0.83**	**4.25**	**0.84**	**4.27**	**0.84**	**0.440**
		**ACAT4**	**3.99**	**1.28**	**3.85**	**1.14**	**3.89**	**1.18**	**0.355**
		**ACAT5**	**4.35**	**0.84**	**4.26**	**0.81**	**4.28**	**0.82**	**0.413**
		**ACAT6**	**3.98**	**1.26**	**3.92**	**1.06**	**3.94**	**1.12**	**0.703**
		**ALL**	**4.17**	**1.07**	**4.08**	**0.97**	**4.10**	**1.00**	**0.101**
	**Biofabrication components for advanced therapies**	**BCAT1**	**4.51**	**0.75**	**4.40**	**0.79**	**4.43**	**0.78**	**0.260**
		**BCAT2**	**4.55**	**0.65**	**4.34**	**0.77**	**4.40**	**0.74**	**0.029**
		**BCAT3**	**4.40**	**0.79**	**4.13**	**0.99**	**4.21**	**0.95**	**0.026**
		**BCAT4**	**4.30**	**0.85**	**4.06**	**1.03**	**4.12**	**0.99**	**0.057**
		**BCAT5**	**4.25**	**0.88**	**4.12**	**0.99**	**4.15**	**0.96**	**0.279**
		**BCAT6**	**4.23**	**0.91**	**4.13**	**0.99**	**4.15**	**0.97**	**0.416**
		**ALL**	**4.37**	**0.81**	**4.19**	**0.94**	**4.24**	**0.91**	7.08E-05*
	**Centers for biofabrication and storage of advanced therapies**	**CBSAT1**	**4.38**	**0.83**	**4.29**	**0.81**	**4.32**	**0.82**	**0.396**
		**CBSAT2**	**4.43**	**0.70**	**4.28**	**0.82**	**4.32**	**0.79**	**0.149**
		**CBSAT3**	**4.15**	**1.00**	**4.13**	**0.96**	**4.13**	**0.97**	**0.812**
		**CBSAT4**	**3.80**	**1.17**	**3.60**	**1.15**	**3.65**	**1.15**	**0.177**
		**CBSAT5**	**3.05**	**1.43**	**2.72**	**1.23**	**2.81**	**1.30**	**0.047**
		**CBSAT6**	**4.27**	**0.91**	**4.23**	**0.86**	**4.24**	**0.88**	**0.708**
		**ALL**	**4.01**	**1.14**	**3.87**	**1.14**	**3.91**	**1.14**	**0.020**
	**PROCEDURAL COMPONENT**	** **	**4.21**	**1.01**	**4.09**	**0.99**	**4.12**	**1.00**	3.89E-06*

Statistically significant values (p<0.00017) are highlighted with asterisks (*).

**Table 4:** Mean values and standard deviations (SD) for the attitudinal component and for each topic and each item included in this component. In each case, results are shown for male and female residents separately, and for all residents together. The last column shows the statistical p value for ANOVA comparisons between genders.

COMPONENT	TOPIC	ITEM	MEAN MALES	SD MALES	MEAN FEMALES	SD FEMALES	MEAL ALL RESIDENTS	SD ALL RESIDENTS	MALES VS. FEMALES ANOVA p VALUE
**ATTITUDINAL**	**Research interest in advanced therapies**	**RIAT1**	**3.85**	**1.20**	**3.83**	**1.05**	**3.83**	**1.09**	**0.907**
		**RIAT2**	**3.90**	**1.16**	**3.86**	**1.07**	**3.87**	**1.09**	**0.732**
		**RIAT3**	**3.92**	**1.16**	**3.79**	**1.09**	**3.82**	**1.11**	**0.364**
		**RIAT4**	**4.25**	**0.92**	**4.23**	**0.76**	**4.23**	**0.80**	**0.823**
		**RIAT5**	**4.33**	**0.84**	**4.17**	**0.82**	**4.21**	**0.83**	**0.119**
		**RIAT6**	**4.36**	**0.90**	**4.11**	**0.91**	**4.18**	**0.91**	**0.036**
		**ALL**	**4.10**	**1.06**	**4.00**	**0.97**	**4.03**	**1.00**	**0.053**
	**Research interest in classical therapies**	**RICT1**	**3.65**	**1.32**	**3.63**	**1.25**	**3.64**	**1.27**	**0.878**
		**RICT2**	**3.81**	**1.21**	**3.77**	**1.08**	**3.78**	**1.12**	**0.776**
		**RICT3**	**3.69**	**1.28**	**3.84**	**1.07**	**3.80**	**1.13**	**0.311**
		**RICT4**	**3.70**	**1.21**	**4.00**	**1.04**	**3.91**	**1.09**	**0.037**
		**RICT5**	**4.29**	**0.95**	**4.18**	**0.85**	**4.21**	**0.88**	**0.352**
		**RICT6**	**4.21**	**0.97**	**4.22**	**0.81**	**4.22**	**0.86**	**0.943**
		**ALL**	**3.89**	**1.19**	**3.94**	**1.05**	**3.93**	**1.09**	**0.445**
	**Valuation of centers for advanced therapies**	**VCAT1**	**3.81**	**1.04**	**3.74**	**1.05**	**3.76**	**1.04**	**0.609**
		**VCAT2**	**3.90**	**1.03**	**3.86**	**1.02**	**3.87**	**1.02**	**0.740**
		**VCAT3**	**3.87**	**0.98**	**3.86**	**1.00**	**3.86**	**1.00**	**0.922**
		**VCAT4**	**3.90**	**0.99**	**3.83**	**0.99**	**3.85**	**0.99**	**0.552**
		**VCAT5**	**3.69**	**1.13**	**3.62**	**1.11**	**3.64**	**1.12**	**0.604**
		**VCAT6**	**2.54**	**1.29**	**2.30**	**1.18**	**2.37**	**1.22**	**0.133**
		**ALL**	**3.62**	**1.18**	**3.53**	**1.20**	**3.56**	**1.19**	**0.172**
	**Valuation of treatment with advanced therapies**	**VTAT1**	**4.29**	**0.82**	**4.32**	**0.79**	**4.31**	**0.80**	**0.709**
		**VTAT2**	**4.12**	**1.03**	**4.25**	**0.84**	**4.21**	**0.90**	**0.273**
		**VTAT3**	**4.32**	**0.75**	**4.27**	**0.84**	**4.29**	**0.82**	**0.646**
		**VTAT4**	**4.10**	**1.03**	**4.05**	**0.96**	**4.06**	**0.98**	**0.698**
		**VTAT5**	**4.35**	**0.80**	**4.33**	**0.76**	**4.33**	**0.77**	**0.868**
		**VTAT6**	**4.31**	**0.73**	**4.28**	**0.81**	**4.29**	**0.78**	**0.753**
		**ALL**	**4.25**	**0.87**	**4.25**	**0.84**	**4.25**	**0.85**	**0.944**
	**ATTITUDINAL COMPONENT**	** **	**3.96**	**1.11**	**3.93**	**1.05**	**3.94**	**1.07**	**0.220**

**Fig 1:**
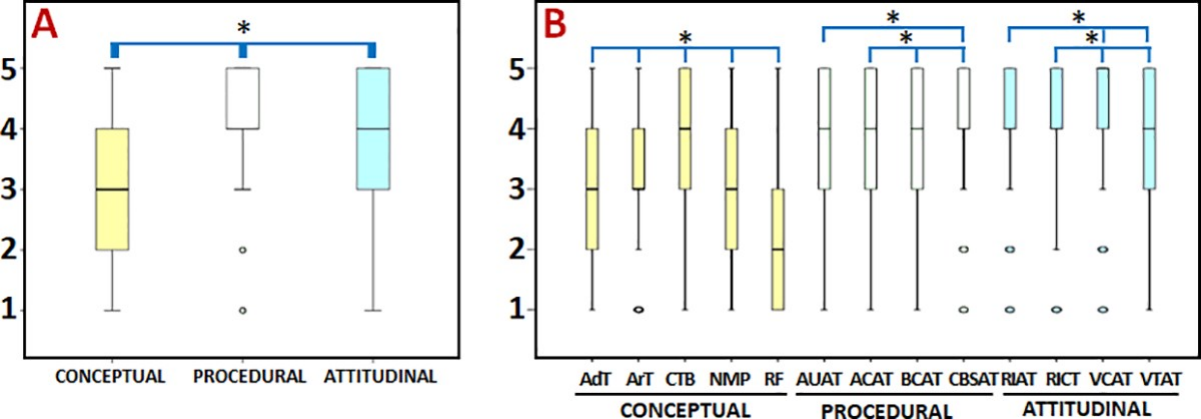
Boxplots of the results for the components and topics analyzed in this study. A: Components. B: Topics. Statistically significant differences (p<0.00017) are labeled with asterisks (*).

First, we analyzed the results for the three components evaluated in the questionnaire. Comparisons across the different components showed that the highest scores appeared in the procedural component (average 4.12±1.00), followed by the attitudinal component (3.94±1.07) and the conceptual component (3.04±1.43). Averages and standard deviations are shown in Tables 2–4 and Fig 1A. Tables 2–4 show the statistical p values for the comparison of males vs. females. In this regard, we found that average scores were significantly higher for males than for females only for the procedural component (Table 3).

On the other hand, we compared the three components -conceptual, procedural and attitudinal- using the ANOVA test. Results showed statistical differences among the three components, with all comparisons being statistically significant (p<0.00017): conceptual vs. procedural, conceptual vs. attitudinal and procedural vs. attitudinal (Fig 1A).

Second, we analyzed the topics. Analysis of the topics in the conceptual component showed the highest scores for the “Cell and tissue basis of the human body” topic, whereas the lowest scores were found for the “Regulatory framework” topic (Table 2 and Fig 1B). Table 2 shows the statistical p values for the ANOVA test for males vs. females. No gender differences were found.

When each of the 5 topics in the conceptual component was compared with the other topics in this component, the differences were statistically significant (p<0.00017) for all comparisons (Fig 1B).

For the procedural component, the highest scores were found for the “Biofabrication components for advanced therapies” topic, whereas the lowest were found for “Centers for biofabrication and storage of advanced therapies” (Table 3 and Fig 1B). For gender comparisons, we found that males assigned higher scores than females in items under the “Biofabrication components for advanced therapies” topic (statistically significant differences) (Table 3).

Pairwise comparisons of these topics revealed statistically significant differences (p<0.00017) for all comparisons except “Application and use of advanced therapies” vs. “Biofabrication components for advanced therapies”, and “Application and use of advanced therapies” vs. “Application center for advanced therapies”, which were nonsignificant (Fig 1B).

When topics included in the attitudinal component were analyzed, we found that the highest scores corresponded to the “Valuation of treatment with advanced therapies” topic, while the lowest scores were found for “Valuation of centers for advanced therapies” (Table 4 and Fig 1B). No differences were observed between males and females for any of the topics (Table 4).

Pairwise comparisons between specific topics in this component yielded statistically significant differences (p<0.00017) among all topics, except for the comparison between “Research interest in advanced therapies” vs. “Research interest in classical therapies” (Fig 1B).

**Fig 2:**
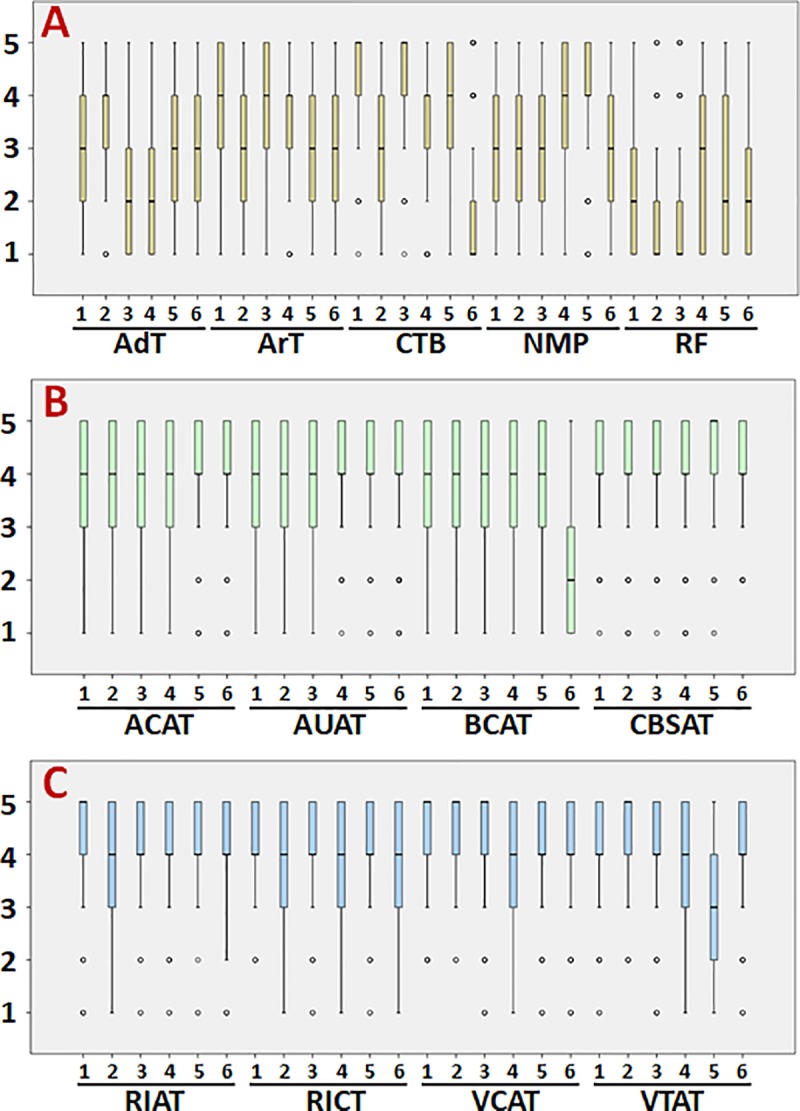
Boxplots of the results for the items included in each topic. A: Items in the conceptual component. B: Items in the procedural component. C: Items in the attitudinal component.

Third, we analyzed the results for each specific item. Averages and standard deviations for each item are shown in Table 2, whereas the statistical p values for the comparison of two specific items are shown in Table 5. In the conceptual component (Fig 2A), we did not found any significant differences between male residents and female residents. For the “Advanced therapies” topic, the highest scores were given to item AdT2 “Are you familiar with the concept of gene therapy?”, which showed statistically significant differences (p<0.00017) compared to the rest of the items in this topic (Table 5). In contrast, items AdT3 “Are you familiar with the concept of somatic cell therapy?” and AdT4 “Are you familiar with the concept of combined advanced therapy?” received significantly lower scores than the remaining items under this topic. For the “Artificial tissues” topic we found the highest score for item ArT3 “Are you familiar with the concept of growth factors?”, which was significantly higher than the remaining item scores, whereas ArT2 “Are you familiar with the concept of biomaterial?”, ArT5 “Are you familiar with the concept of tissue engineering?” and ArT6 “Are you familiar with the concept of regenerative medicine?” had the lowest scores. For the “Cell and tissue basis of the human body” topic, items CTB1 “Are you familiar with the concept of cell?” and CTB2 Are you familiar with the concept of tissue?” had the highest scores of all items in this topic and in the conceptual component overall. However, item CTB6 “Are you familiar with the concept of IPS cell?” scored significantly lower than the remaining items in this topic. For the “Novel medical products” topic, item NMP5 “Do you know if growth factors are used to treat diseases?” scored significantly higher than the other items, whereas NMP6 “Do you know if there are benefits of these therapies with respect to current treatment techniques?” and NMP3 “Do you know if transplanted organs are medicines?” had the lowest scores. Regarding the “Regulatory framework” topic, items RF4 “Do you know if it is mandatory to perform a clinical trial before using advanced therapy products?” and RF5 “Do you know if all advanced therapies require authorization from the Spanish and European agencies for their implementation?” received significantly higher scores than the other items, whereas the lowest scores were found for RF2 “Do you know what GMP rooms are?” and RF3 “Do you know if it is mandatory to manufacture advanced therapy products considered medicines in GMP rooms?”.

**Table 5:** Statistical p values for pairwise comparisons of two specific items included in each topic of the conceptual component.

Advanced therapies	p value	Artificial tissues	p value	Cell and tissue basis of the human body	p value	Novel medical products	p value	Regulatory framework	p value
**AdT1 vs AdT2**	3.42E-14*	**ArT1 vs ArT2**	1.74E-04	**CTB1 vs CTB2**	0.856	**NMP1 vs NMP2**	0.947	**RF1 vs RF2**	2.59E-11*
**AdT1 vs AdT3**	2.99E-06*	**ArT1 vs ArT3**	6.95E-06*	**CTB1 vs CTB3**	3.87E-02	**NMP1 vs NMP3**	1.05E-02	**RF1 vs RF3**	2.64E-12*
**AdT1 vs AdT4**	2.28E-08*	**ArT1 vs ArT4**	0.972	**CTB1 vs CTB4**	3.05E-17*	**NMP1 vs NMP4**	1.50E-04*	**RF1 vs RF4**	2.02E-13*
**AdT1 vs AdT5**	0.924	**ArT1 vs ArT5**	1.13E-05*	**CTB1 vs CTB5**	1.50E-33*	**NMP1 vs NMP5**	2.30E-25*	**RF1 vs RF5**	1.04E-08*
**AdT1 vs AdT6**	0.873	**ArT1 vs ArT6**	2.53E-04	**CTB1 vs CTB6**	4.30E-156*	**NMP1 vs NMP6**	8.67E-05*	**RF1 vs RF6**	0.193
**AdT2 vs AdT3**	2.19E-33*	**ArT2 vs ArT3**	6.31E-17*	**CTB2 vs CTB3**	0.052	**NMP2 vs NMP3**	1.28E-02	**RF2 vs RF3**	0.769
**AdT2 vs AdT4**	1.31E-38*	**ArT2 vs ArT4**	1.01E-04*	**CTB2 vs CTB4**	3.41E-17*	**NMP2 vs NMP4**	1.18E-04*	**RF2 vs RF4**	1.68E-40*
**AdT2 vs AdT5**	7.18E-13*	**ArT2 vs ArT5**	0.530	**CTB2 vs CTB5**	1.12E-33*	**NMP2 vs NMP5**	1.38E-25*	**RF2 vs RF5**	3.14E-32*
**AdT2 vs AdT6**	7.91E-13*	**ArT2 vs ArT6**	0.921	**CTB2 vs CTB6**	7.63E-158*	**NMP2 vs NMP6**	1.15E-04*	**RF2 vs RF6**	1.59E-15*
**AdT3 vs AdT4**	0.383	**ArT3 vs ArT4**	4.06E-06*	**CTB3 vs CTB4**	3.02E-11*	**NMP3 vs NMP4**	4.56E-10*	**RF3 vs RF4**	2.60E-42*
**AdT3 vs AdT5**	4.86E-06*	**ArT3 vs ArT5**	1.68E-19*	**CTB3 vs CTB5**	1.27E-25*	**NMP3 vs NMP5**	5.51E-37*	**RF3 vs RF5**	7.58E-34*
**AdT3 vs AdT6**	3.08E-06*	**ArT3 vs ArT6**	1.36E-16*	**CTB3 vs CTB6**	1.22E-142*	**NMP3 vs NMP6**	0.156	**RF3 vs RF6**	1.02E-16*
**AdT4 vs AdT5**	5.25E-08*	**ArT4 vs ArT5**	5.57E-06*	**CTB4 vs CTB5**	3.23E-05*	**NMP4 vs NMP5**	6.56E-11*	**RF4 vs RF5**	0.103
**AdT4 vs AdT6**	2.94E-08*	**ArT4 vs ArT6**	1.50E-04*	**CTB4 vs CTB6**	8.36E-92*	**NMP4 vs NMP6**	8.03E-14*	**RF4 vs RF6**	7.02E-10*
**AdT5 vs AdT6**	0.951	**ArT5 vs ArT6**	0.467	**CTB5 vs CTB6**	4.26E-63*	**NMP5 vs NMP6**	6.01E-43*	**RF5 vs RF6**	6.25E-06*

Statistically significant values (p<0.00017) are highlighted with asterisks (*).

Analysis of the items in the procedural component (Fig 2B) disclosed some significant differences. Averages and standard deviations for each item are shown in Table 3, and the statistical p values for the comparison of two specific items are shown in Table 6. When males and females were compared, we did not found any significant differences. In the “Application and use of advanced therapies” topic, item AUAT1 “Would you use the patient's own cells for treatment with cell therapy?” scored significantly higher (p<0.00017; Table 6) than item AUAT2 “Would you use cells from donors to treat a patient with cell therapy?”. Item AUAT2 “Would you use cells from donors to treat a patient with cell therapy?” scored significantly lower than AUAT3 “Would you apply cell therapy to treat a disease?”, AUAT4 “Would you apply gene therapy to treat a disease?” and AUAT5 “Would you apply tissue-engineered tissues to treat a disease?”. Regarding the “Application center for advanced therapies” topic, the three items that mentioned hospitals, e.g. ACAT1 “Would you use hospitals for a cell therapy treatment?”, ACAT3 “Would you use hospitals for a gene therapy treatment?” and ACAT5 “Would you use hospitals to treat a patient with artificial tissues generated by tissue engineering?” received significantly higher scores than the three items on advanced therapies in primary care health centers (ACAT2, ACAT4 and ACAT6). For the “Biofabrication components for advanced therapies” topic, the highest scores were found for items BCAT1 “Would you use umbilical cord stem cells to build artificial tissues?” and BCAT2 “Would you use bone marrow stem cells to build artificial tissues?”. The differences were statistically significant for the comparison between the first of these items and BCAT4 “Would you use dental pulp stem cells to build artificial tissues?”, BCAT5 “Would you build artificial tissues with biomaterials?” and BCAT6 “Would you build artificial tissues with growth factors?”. In addition, the difference between BCAT2 “Would you use bone marrow stem cells to build artificial tissues?” and BCAT4 “Would you use dental pulp stem cells to build artificial tissues?” was statistically significant. For the “Centers for biofabrication and storage of advanced therapies” topic, items CBSAT1 “Would you store artificial tissues in tissue banks for deferred use?”, CBSAT2 “Would you store cells in tissue banks for deferred use?” and CBSAT6 “Would you use a research center to build artificial tissues?” obtained the highest scores, whereas CBSAT5 “Would you use a pharmaceutical company to build artificial tissues?” scored lowest of all items across all procedural topics. The differences were statistically significant for, among others, the comparisons of item CBSAT5 “Would you use a pharmaceutical company to build artificial tissues?”, which received the lowest score, and CBSAT4 “Would you use a primary care health center to build artificial tissues?” vs. the remaining items.

**Table 6:** Statistical p values for pairwise comparisons of two specific items included in each topic of the procedural component.

APPLICATION AND USE OF ADVANCED THERAPIES	p value	APPLICATION CENTER FOR ADVANCED THERAPIES	p value	BIOFABRICATION COMPONENTS FOR ADVANCED THERAPIES	p value	CENTERS FOR BIOFABRICATION AND STORAGE OF ADVANCED THERAPIES	p value
**AUAT1 vs AUAT2**	5.75E-09*	**ACAT1 vs ACAT2**	3.93E-05*	**BCAT1 vs BCAT2**	0.608	**CBSAT1 vs CBSAT2**	0.919
**AUAT1 vs AUAT3**	0.123	**ACAT1 vs ACAT3**	0.879	**BCAT1 vs BCAT3**	0.002	**CBSAT1 vs CBSAT3**	0.013
**AUAT1 vs AUAT4**	0.106	**ACAT1 vs ACAT4**	1.49E-06*	**BCAT1 vs BCAT4**	2.95E-05*	**CBSAT1 vs CBSAT4**	2.45E-15*
**AUAT1 vs AUAT5**	0.309	**ACAT1 vs ACAT5**	1.000	**BCAT1 vs BCAT5**	1.25E-04*	**CBSAT1 vs CBSAT5**	2.22E-53*
**AUAT1 vs AUAT6**	2.61E-04	**ACAT1 vs ACAT6**	1.19E-05*	**BCAT1 vs BCAT6**	1.29E-04*	**CBSAT1 vs CBSAT6**	0.289
**AUAT2 vs AUAT3**	4.39E-06*	**ACAT2 vs ACAT3**	1.04E-04*	**BCAT2 vs BCAT3**	0.007	**CBSAT2 vs CBSAT3**	0.009
**AUAT2 vs AUAT4**	8.26E-06*	**ACAT2 vs ACAT4**	0.434	**BCAT2 vs BCAT4**	1.33E-04*	**CBSAT2 vs CBSAT4**	6.61E-16*
**AUAT2 vs AUAT5**	2.59E-07*	**ACAT2 vs ACAT5**	5.36E-05*	**BCAT2 vs BCAT5**	0.001	**CBSAT2 vs CBSAT5**	1.39E-54*
**AUAT2 vs AUAT6**	0.021	**ACAT2 vs ACAT6**	0.798	**BCAT2 vs BCAT6**	0.001	**CBSAT2 vs CBSAT6**	0.240
**AUAT3 vs AUAT4**	0.920	**ACAT3 vs ACAT4**	4.66E-06*	**BCAT3 vs BCAT4**	0.289	**CBSAT3 vs CBSAT4**	5.33E-08*
**AUAT3 vs AUAT5**	0.567	**ACAT3 vs ACAT5**	0.882	**BCAT3 vs BCAT5**	0.467	**CBSAT3 vs CBSAT5**	2.11E-39*
**AUAT3 vs AUAT6**	0.022	**ACAT3 vs ACAT6**	3.43E-05*	**BCAT3 vs BCAT6**	0.468	**CBSAT3 vs CBSAT6**	0.146
**AUAT4 vs AUAT5**	0.505	**ACAT4 vs ACAT5**	2.18E-06*	**BCAT4 vs BCAT5**	0.707	**CBSAT4 vs CBSAT5**	2.79E-16*
**AUAT4 vs AUAT6**	0.030	**ACAT4 vs ACAT6**	0.595	**BCAT4 vs BCAT6**	0.707	**CBSAT4 vs CBSAT6**	4.76E-12*
**AUAT5 vs AUAT6**	0.004	**ACAT5 vs ACAT6**	1.68E-05*	**BCAT5 vs BCAT6**	1.000	**CBSAT5 vs CBSAT6**	1.03E-47*

Statistically significant values (p<0.00017) are highlighted with asterisks (*).

Finally, in the attitudinal component (Fig 2C), we did not found significant differences between the scores given by males and females (Table 4). Averages and standard deviations for each item are shown in Table 4, and the statistical p values for the comparison of two specific items are shown in Table 7. For the “Research interest in advanced therapies” topic, the highest scores were found for items RIAT4 “Do you think performing clinical trials in cell therapy is a good idea?”, RIAT5 “Do you think performing clinical trials with artificial tissues is a good idea?” and RIAT6 “Do you think performing clinical trials in gene therapy is a good idea?”, and some differences compared to the other items in this topic were statistically significant (p<0.00017; Table 7). Analysis of the “Research interest in classical therapies” topic showed that items RICT5 “Do you think performing clinical trials to test pharmaceutical drugs is a good idea?” and RICT6 “Do you think performing clinical trials in physical therapy is a good idea?” had the highest scores, with significant differences compared to most of the remaining items, whereas the lowest values were seen for item RICT1 “Are you interested in research in surgery?”. Regarding the “Valuation of centers for advanced therapies” topic, we found that items VCAT2 “Do you prefer hospitals for the application of gene therapy?”, VCAT3 “Do you prefer hospitals for the application of artificial tissue therapy?” and VCAT4 “Do you prefer hospitals for the application of combined advanced therapies?” received the highest scores, whereas VCAT6 “Do you prefer artificial tissues to be manufactured by the pharmaceutical industry?” had the lowest item score across all attitudinal topics, with significant differences compared to the rest of the items. In the last attitudinal topic, “Valuation of treatment with advanced therapies”, most items received similarly high scores, whereas item VTAT4 “Do you think so-called advanced therapies are a good idea?” had the lowest score.

**Table 7:** Statistical p values for pairwise comparisons of two specific items included in each topic of the attitudinal component.

RESEARCH INTEREST IN ADVANCED THERAPIES	p value	RESEARCH INTEREST IN CLASSICAL THERAPIES	p value	VALUATION OF CENTERS FOR ADVANCED THERAPIES	p value	VALUATION OF TREATMENT WITH ADVANCED THERAPIES	p value
**RIAT1 vs RIAT2**	0.682	**RICT1 vs RICT2**	0.143	**VCAT1 vs VCAT2**	0.179	**VTAT1 vs VTAT2**	0.136
**RIAT1 vs RIAT3**	0.911	**RICT1 vs RICT3**	0.104	**VCAT1 vs VCAT3**	0.230	**VTAT1 vs VTAT3**	0.686
**RIAT1 vs RIAT4**	4.52E-07*	**RICT1 vs RICT4**	0.004	**VCAT1 vs VCAT4**	0.279	**VTAT1 vs VTAT4**	0.001
**RIAT1 vs RIAT5**	2.08E-06*	**RICT1 vs RICT5**	2.61E-10*	**VCAT1 vs VCAT5**	0.163	**VTAT1 vs VTAT5**	0.755
**RIAT1 vs RIAT6**	2.94E-05*	**RICT1 vs RICT6**	9.67E-11*	**VCAT1 vs VCAT6**	9.34E-44*	**VTAT1 vs VTAT6**	0.680
**RIAT2 vs RIAT3**	0.604	**RICT2 vs RICT3**	0.856	**VCAT2 vs VCAT3**	0.871	**VTAT2 vs VTAT3**	0.274
**RIAT2 vs RIAT4**	4.42E-06*	**RICT2 vs RICT4**	0.140	**VCAT2 vs VCAT4**	0.776	**VTAT2 vs VTAT4**	0.051
**RIAT2 vs RIAT5**	1.76E-05*	**RICT2 vs RICT5**	2.19E-07*	**VCAT2 vs VCAT5**	0.007	**VTAT2 vs VTAT5**	0.071
**RIAT2 vs RIAT6**	1.83E-04	**RICT2 vs RICT6**	9.04E-08*	**VCAT2 vs VCAT6**	2.05E-50*	**VTAT2 vs VTAT6**	0.265
**RIAT3 vs RIAT4**	2.99E-07*	**RICT3 vs RICT4**	0.200	**VCAT3 vs VCAT4**	0.902	**VTAT3 vs VTAT4**	0.002
**RIAT3 vs RIAT5**	1.39E-06*	**RICT3 vs RICT5**	7.45E-07*	**VCAT3 vs VCAT5**	0.010	**VTAT3 vs VTAT5**	0.473
**RIAT3 vs RIAT6**	2.02E-05*	**RICT3 vs RICT6**	3.24E-07*	**VCAT3 vs VCAT6**	1.63E-50*	**VTAT3 vs VTAT6**	1.000
**RIAT4 vs RIAT5**	0.765	**RICT4 vs RICT5**	2.69E-04	**VCAT4 vs VCAT5**	0.014	**VTAT4 vs VTAT5**	1.63E-04*
**RIAT4 vs RIAT6**	0.449	**RICT4 vs RICT6**	1.46E-04*	**VCAT4 vs VCAT6**	4.51E-50*	**VTAT4 vs VTAT6**	0.002
**RIAT5 vs RIAT6**	0.641	**RICT5 vs RICT6**	0.888	**VCAT5 vs VCAT6**	1.20E-35*	**VTAT5 vs VTAT6**	0.463

Statistically significant values (p<0.00017) are highlighted with asterisks (*).

## Discussion

The increasing relevance of gene-, cell- and tissue-based therapies in medicine and the expectations these novel therapies generate in the population of health care users require specific studies in this field. Incorporating knowledge about advanced therapies in training programs for future family medicine physicians will allow these professionals to contribute effectively to health education in the user population. However, before training activities are developed and implemented for family medicine residents, their conceptual, procedural and attitudinal profiles related to these therapies should be investigated. As indicated by different theoretical frameworks in educational research, a wide range of circumstances can potentially promote or limit the learning process in a particular situation [20–22]. Moreover, studies of medical residents’ perceptions regarding professional practice have demonstrated the importance that residents give to the need to overcome shortcomings in their professional behavior, cognitive ability and procedure skills [23, 24].

In the present study we examined how family medicine residents perceive advanced therapies in terms of their knowledge, their approach to implementing these therapies, and their attitudes toward these novel therapeutic tools. According to the overall results of this study, the scores for the procedural component were the highest among the three components of perception in family medicine residents, whereas the lowest scores were found for the conceptual component. Strikingly, although residents perceived that procedures for implementing advanced therapies are well established, probably due to the strict regulation of protocols [1, 25], they also perceived that their cognitive background is insufficient to respond to the expectations generated by these new therapeutic tool.

The scores for the attitudinal component were intermediate between the procedural and conceptual components. Attitudes represent a summation of thinking, emotions and feelings about a specific issue–for example, in the context of learning a course [19]. This facilitates not only personal equilibrium, but also the positive coexistence of professional and social values and beliefs. As pointed out by Li et al. [26], the current literature lacks a discussion of the attitudes of the medical community regarding ways to balance cost-effectiveness with equity in the use of and access to treatments. Our results reflect this situation. In fact, the attitudes of family medicine residents who participated in the present study are situated in a prudent balance between what they perceive could be implemented clinically–the procedural component–and what they perceive they know about advances therapies–the conceptual component.

When we analyzed the different topics included in the conceptual component, our results highlighted that residents gave the highest values to their perceived knowledge of cells and tissues in the human body, and the lowest values to their knowledge of the regulatory framework for these therapies. After knowledge of cells and tissues, the perception of topics regarding knowledge of artificial tissues, advanced therapies and new medical products received progressively lower scores. These results may be explained by the fact that most of the topics and items our respondents scored highest correspond to concepts that are part of the core curriculum of medical training in medical schools [20, 27], in contrast with other concepts such as GMP facilities, IPs cells or biomaterials. In addition, these findings may have been influenced by the widespread use of growth factors among commonly used advanced therapies [28–30], the wide dissemination of news about gene therapy in the lay media [31, 32], and finally, the application to advanced therapies of regulations and protocols that residents are usually familiar with for ordinary therapies (e.g. clinical trials, authorizations, etc.) [33, 34].

Regarding the topics and items included in the procedural component, the highest and lowest scores were found, respectively, for "biofabrication components for advances therapies" and "centers for biofabrication and storage of advances therapies”. In the former case, there was good correlation with the results for the conceptual component, since the topic with the highest score in this component (knowledge of cells and tissues) constitutes the foundation of knowledge elements that are needed for the biofabrication of ATMPs [1, 25]. In the latter case, procedural knowledge referred to centers for the manufacture and storage of advanced therapies is mostly absent from the training objectives for family medicine residents. These results show that residents are better informed about the procedures needed to apply advanced therapies than about the types of center where each therapy should be applied. This is consistent with the low scores obtained for knowledge of the regulatory framework in the conceptual component, as discussed above. Spanish family medicine residents are willing to consider advanced therapies as a possible tool in the therapeutic management of their patients. However, as deduced from their responses, they seem highly aware of the risks and limitations, given that they indicated their preference for using tools that have been well tested in clinical research, e.g. stem cells from the bone marrow or umbilical cord, and would prefer to use autologous cells instead of donor cells. Nevertheless, this situation is likely to change in the immediate future because some stem cells, which can be cryopreserved and stored in tissue banks, are increasingly used in advanced therapies to treat a variety of oncologic, genetic, hematologic and immune deficiency disorders [35–38]. This means that in the foreseeable future, family medicine physicians will come to play an important role not only in patients’ education–i.e., how to guide patients contemplating unproven and unapproved advanced therapies, and thus avoid medical tourism–but also in patients’ safety at the primary care level, in the daily follow-up of patients treated with this type of therapy [39]. Although some authors have expressed alarm over the decrease in the scope of care being provided by family medicine physicians, the emergence of advances therapies opens new areas of activity within the remit of these professionals–activities, however, that the residents in this study did not seem to significantly perceive at the present time [27, 40, 41].

When topics and items of the attitudinal component were analyzed, we found that the highest scores corresponded to valuation of treatment with advanced therapies, while the lowest scores were seen for valuation of centers where these therapies would be implemented. Again, and in consonance with the tendency observed for the procedural component, residents’ attitude-related responses attributed more value to the possible use of these new treatments than to the logistic support that makes these treatments possible. However, they expressed a preference for hospitals as the most appropriate setting for the application of advanced therapies, and were less supportive of the manufacture of artificial tissues as medical products by the pharmaceutical industry. In addition, residents valued research in advanced therapies more highly than research in classical therapies. Although it has been suggested that family medicine residents are less interested in research than other graduates, our results showed that they not only had a positive attitude towards new therapies, but also expressed a more favorable attitude towards research in new therapies than towards research in classical therapies [42, 43]. The attitudes reported by the residents in our sample again showed that they are aware of the need to use proven therapeutic tools for both classical and advanced therapies. This finding argues very strongly in favor of efforts to involve family medicine physicians in health education programs for these new therapies. Family medicine professionals should act as qualified medical educators able to guide patients regarding proven and unproven advanced interventions, and to advise patients on cost-effectiveness in the use of and access to treatments [26, 40, 41, 44, 45].

Regarding gender, our results showed very few differences between male and female residents. Significant differences were found only for the procedural component and for the “Biofabrication components for advanced therapies” topic, but not for the conceptual and attitudinal components. These findings are consistent with the patterns usually described in relation to gender and medicine [15, 46–48]. Our results can contribute to a better understanding of residents’ profiles regarding advanced therapies, and the slight gender differences detected in our respondents should be taken into account when new training programs are implemented in this area. Although differences between genders remain poorly understood, they may result from factors such as role modeling and socialization by family, teachers, peers and the media, rather than from “innate or natural differences” between women and men [48, 49].

In conclusion, this questionnaire-based study provides evidence that can be used to establish profiles associated with the conceptual, procedural and attitudinal components of Spanish family medicine residents’ views on advanced therapies. Although they perceived that procedures to implement advanced therapies are well established, especially in terms of application, they feel their cognitive background is not strong enough to efficiently respond to the expectations generated by these new therapeutic tools, and perceive themselves to be especially underprepared regarding their knowledge of the regulatory framework. In their attitudinal responses, residents gave more value to the possible use of these new treatments than to the logistic support that makes these new therapies possible, and on a secondary level, they also gave more value to research in advanced therapies than in classical therapies. Their keen awareness of the risks and limitations of these treatments was reflected by their preference for using cells that have undergone thorough clinical testing. Although they appropriately situated treatment with these therapies at the hospital level, it is important to note that they significantly associated biofabrication with research centers, although these therapeutic tools can also be produced at different types of facilities.

One of the limitations of this study is the use of a questionnaire designed and validated originally in the Spanish language. Although the most relevant concepts are accurately translatable into English, care should be exercised before using the translated questionnaire for native English speakers, since some items may not perfectly match the answers provided by the Likert-like scale used here. Future work should aim to ensure the accurate translation and cultural adaptation of the questionnaire for respondents in specific settings. Versions of the questionnaire in other languages should be used only after an appropriately validated translation process. Another limitation is the use of the same response scale for all conceptual, procedural and attitudinal items, some of which may require specific answer options. However, using the same scale favored homogeneity and comparability among the items, topics and components.

Despite these limitations, we conclude that the results of this study are potentially useful to support the design of future training programs and health policies of family medicine residents, in view of the rapid development of these treatments, the expectations they raise in patients, and the responsibility of professionals to use these complex therapies in an equitable and cost-effective manner.

## Supporting information

S1 File Raw data showing the scores assigned by each resident to each item of the questionnaire. (XLSX)

S1 Table Questionnaire used in the present study, with the different components, topics, items and abbreviated item names used in the tables. (PDF)
