# Supplementary material for: Evaluation of the awareness of novel advanced therapies among family medicine residents in Spain
Source: PLoS One. 2019 Apr 3;14(4):e0214950. doi: 10.1371/journal.pone.0214950 (PMC6447282; doi:10.1371/journal.pone.0214950)
Supplement: S1 Table — (PDF) [file pone.0214950.s002.pdf]

COMPONENT	TOPIC	ITEM	ITEM	1= strongly disagree	2= disagree	3= neither agree nor disagree	4= agree	5= strongly agree
CONCEPTUAL	Advanced therapies	AdT1	Do you know what advanced therapies are?					
		AdT2	Are you familiar with the concept of gene therapy?					
		AdT3	Are you familiar with the concept of somatic cell therapy?					
		AdT4	Are you familiar with the concept of combined advanced therapy?					
		AdT5	Do you know if gene therapy is an advanced therapy?					
		AdT6	Do you know if tissue engineering is an advanced therapy?					
	Artificial tissues	ArT1	Are you familiar with the concept of artificial tissue?					
		ArT2	Are you familiar with the concept of biomaterial?					
		ArT3	Are you familiar with the concept of growth factors?					
		ArT4	Do you distinguish conceptually between a natural tissue and an artificial one?					
		ArT5	Are you familiar with the concept of tissue engineering?					
		ArT6	Are you familiar with the concept of regenerative medicine?					
	Cell and tissue basis of the human body	CTB1	Are you familiar with the concept of cell?					
		CTB2	Are you familiar with the concept of tissue?					
		CTB3	Are you familiar with the concept of stem cell?					
		CTB4	Are you familiar with the concept of embryonic stem cell?					
		CTB5	Are you familiar with the concept of adult stem cell?					
		CTB6	Are you familiar with the concept of IPS cell?					
	Novel medical products	NMP1	Do you know if a cell can be considered a medicine?					
		NMP2	Do you know if a tissue can be considered a medicine?					
		NMP3	Do you know if transplanted organs are medicines?					
		NMP4	Do you know if biomaterials are used to treat diseases?					
		NMP5	Do you know if growth factors are used to treat diseases?					
		NMP6	Do you know if there are benefits of these therapies with respect to current treatment techniques?					
	Regulatory frame	RF1	Do you know if there is specific EU legislation for advanced therapies?					
		RF2	Do you know what GMP rooms are?					
		RF3	Do you know if it is mandatory to manufacture advanced therapy products considered medicines in GMP rooms?					
		RF4	Do you know if it is mandatory to perform a clinical trial before using advanced therapy products?					
		RF5	Do you know if all advanced therapies require authorization from the Spanish and European agencies for their implementation?					
		RF6	Do you know if advanced therapies are in the service portfolio of the National Health System?					
PROCEDURAL	Application and use of advanced therapies	AUAT1	Would you use the patient's own cells for treatment with cell therapy?					
		AUAT2	Would you use cells from donors to treat a patient with cell therapy?					
		AUAT3	Would you apply cell therapy to treat a disease?					
		AUAT4	Would you apply gene therapy to treat a disease?					
		AUAT5	Would you apply tissue-engineered tissues to treat a disease?					
		AUAT6	Would you apply artificial tissues built with cells, biomaterials and growth factors together to treat a disease?					
	Application center for advanced therapies	ACAT1	Would you use hospitals for a cell therapy treatment?					
		ACAT2	Would you use primary care health centers to monitor patients treated with cell therapy?					
		ACAT3	Would you use hospitals for a gene therapy treatment?					
		ACAT4	Would you use primary care health centers to follow up patients treated with gene therapy?					
		ACAT5	Would you use hospitals to treat a patient with artificial tissues generated by tissue engineering?					
		ACAT6	Would you use primary care health centers to monitor patients treated with artificial tissues generated by tissue engineering?					
	Biofabrication components for advanced therapies	BCAT1	Would you use umbilical cord stem cells to build artificial tissues?					
		BCAT2	Would you use bone marrow stem cells to build artificial tissues?					
		BCAT3	Would you use adipose tissue stem cells to build artificial tissues?					
		BCAT4	Would you use dental pulp stem cells to build artificial tissues?					
		BCAT5	Would you build artificial tissues with biomaterials?					
		BCAT6	Would you build artificial tissues with growth factors?					
	Centers for biofabrication and storage of advanced therapies	CBSAT1	Would you store artificial tissues in tissue banks for deferred use?					
		CBSAT2	Would you store cells in tissue banks for deferred use?					
		CBSAT3	Would you store genes in tissue banks for deferred use?					
		CBSAT4	Would you use a primary care health center to build artificial tissues?					
		CBSAT5	Would you use a pharmaceutical company to build artificial tissues?					
		CBSAT6	Would you use a research center to build artificial tissues?					
ATTITUDINAL	Research interest in advanced therapies	RIAT1	Are you interested in cell therapy research?					
		RIAT2	Are you interested in gene therapy research?					
		RIAT3	Are you interested in artificial tissue therapy research?					
		RIAT4	Do you think clinical trials in cell therapy are a good idea?					
		RIAT5	Do you think clinical trials with artificial tissues are a good idea?					
		RIAT6	Do you think clinical trials in gene therapy are a good idea?					
	Research interest in classical therapies	RICT1	Are you interested in research in surgery?					
		RICT2	Are you interested in pharmacotherapy research?					
		RICT3	Are you interested in research in physical medicine and physiotherapy?					
		RICT4	Are you interested in psychotherapy research?					
		RICT5	Do you think clinical trials to test pharmaceutical drugs are a good idea?					
		RICT6	Do you think clinical trials in physical therapy are a good idea?					
	Valuation of centers for advanced therapies	VCAT1	Do you prefer hospitals for the application of cell therapy?					
		VCAT2	Do you prefer hospitals for the application of gene therapy?					
		VCAT3	Do you prefer hospitals for the application of artificial tissue therapy?					
		VCAT4	Do you prefer hospitals for the application of combined advanced therapies?					
		VCAT5	Do you prefer artificial tissues to be manufactured in hospitals?					
		VCAT6	Do you prefer artificial tissues to be manufactured by the pharmaceutical industry?					
	Valuation on treatment with advanced therapies	VTAT1	Do you think cell therapy is a good idea?					
		VTAT2	Do you think gene therapy is a good idea?					
		VTAT3	Do you think therapy with artificial tissues is a good idea?					
		VTAT4	Do you think so-called advanced therapies is a good idea?					
		VTAT5	Do you think therapy with physical medicine is a good idea?					
		VTAT6	Do you think pharmaceutical drug therapy is a good idea?					
